# Cost-consequence analysis of remifentanil-based analgo-sedation vs. conventional analgesia and sedation for patients on mechanical ventilation in the Netherlands

**DOI:** 10.1186/cc9313

**Published:** 2010-11-01

**Authors:** Maiwenn J Al, Leona Hakkaart, Siok Swan Tan, Jan Bakker

**Affiliations:** 1 Institute for Medical Technology Assessment, Erasmus University, Burg. Oudlaan 50, Rotterdam, 3062 PA, The Netherlands; 2 Department of Intensive Care, Erasmus MC University Medical Centre, Dr. Molewaterplein 50, Rotterdam, 3015 GE, The Netherlands

## Abstract

**Introduction:**

Hospitals are increasingly forced to consider the economics of technology use. We estimated the incremental cost-consequences of remifentanil-based analgo-sedation (RS) vs. conventional analgesia and sedation (CS) in patients requiring mechanical ventilation (MV) in the intensive care unit (ICU), using a modelling approach.

**Methods:**

A Markov model was developed to describe patient flow in the ICU. The hourly probabilities to move from one state to another were derived from UltiSAFE, a Dutch clinical study involving ICU patients with an expected MV-time of two to three days requiring analgesia and sedation. Study medication was either: CS (morphine or fentanyl combined with propofol, midazolam or lorazepam) or: RS (remifentanil, combined with propofol when required). Study drug costs were derived from the trial, whereas all other ICU costs were estimated separately in a Dutch micro-costing study. All costs were measured from the hospital perspective (price level of 2006). Patients were followed in the model for 28 days. We also studied the sub-population where weaning had started within 72 hours.

**Results:**

The average total 28-day costs were €15,626 with RS versus €17,100 with CS, meaning a difference in costs of €1474 (95% CI -2163, 5110). The average length-of-stay (LOS) in the ICU was 7.6 days in the RS group versus 8.5 days in the CS group (difference 1.0, 95% CI -0.7, 2.6), while the average MV time was 5.0 days for RS versus 6.0 days for CS. Similar differences were found in the subgroup analysis.

**Conclusions:**

Compared to CS, RS significantly decreases the overall costs in the ICU.

**Trial Registration:**

Clinicaltrials.gov NCT00158873.

## Introduction

The vast majority of patients admitted to the intensive care unit (ICU) requires mechanical ventilation. In order to facilitate mechanical ventilation these patients often require the administration of both analgesics (often opioids) and sedatives [1]. This combination is applied to control pain, relieve agitation and anxiety, aid compliance to the mechanical ventilator, and, hence, to maintain comfort. However, when administered for a longer period, the pharmacodynamic effects of conventional opioids such as fentanyl and morphine become unpredictable and are often prolonged as a result of re-distribution and accumulation [2]. This may increase the risk of suppressed respiratory drive and potentially delay weaning and extend the duration of mechanical ventilation.

Decreasing the duration of mechanical ventilation might lead to medical and economic benefits: a shorter mechanical ventilation duration decreases the risk of ventilator-associated morbidity, for example, complications caused by loss of airway defense mechanisms such as nosocomial pneumonia [3-5]. Reduction of the duration of mechanical ventilation may also yield savings in terms of reduced ICU and hospital length of stay and reduced costs [6].

Remifentanil is a selective μ-opioid receptor agonist. It has a rapid onset of action and a short half-time of approximately four minutes, without accumulation after prolonged infusion [7-9]. It can be used as the main drug to provide patient comfort, while the use of the sedative agent is kept to a minimum. However, remifentanil is also more expensive than commonly used sedatives and analgesics.

Predicting the duration of mechanical ventilation and ICU stay can be difficult and the use of long-acting sedatives/analgesics in the early phase of ICU admission can prolong the duration of mechanical ventilation when a patient recovers more quickly than expected [10]. In such unpredictable circumstances, a short-acting agent may improve the speed of weaning from the ventilator and advance ICU discharge. Therefore, UltiSAFE, a trial in a 'real-life' setting was done to compare the duration of mechanical ventilation, weaning time, ICU length of stay, efficacy, and safety of a remifentanil-based analgesia and sedation regimen to conventional sedation and analgesia regimens in a mixed group of medical and post-surgical ICU patients with anticipated short-term (two to three days) mechanical ventilation following the start of the study medication [11]. The latter criterion was based on the fact that remifentanil is only approved for the provision of analgesia in mechanically ventilated ICU patients up to three days [12].

The questions that may be raised are (1) whether remifentanil-based sedation might lead to a shorter MV and ICU length-of-stay (LOS) and (2) whether the higher costs of remifentanil are offset by the cost reduction due to the potentially decreased ICU length of stay. A reduction in ventilator days may lead to cost reduction as the costs per ICU day are up to 30% higher for patients on MV [13]. Therefore, we conducted a cost-consequence study comparing the costs of remifentanil-based sedation (RS) versus conventional-based sedation (CS) in ICU patients requiring mechanical ventilation. Because a large number of patients was still on ventilation after 10 days (20% CS group, 8% RS group) and subsequently censored, we developed a model extrapolating beyond 10 days MV in order to compare the two sedation regimens. We assessed the cost-consequences both for the whole patient population and the on-label subpopulation where weaning had started within 72 hours.

## Materials and methods

### Material/data

Input for the model was derived from UltiSAFE, a Dutch open label, centre-randomized, centre-crossover trial that was conducted at 15 Dutch university and other medical centres between 2004 and 2005. Patients admitted to an ICU with an expected MV-time of two to three days were included [11]. They were randomized to receive conventional sedation (*n *= 109), that is, morphine or fentanyl combined with propofol, midazolam or lorazepam according to Dutch guidelines, or remifentanil-based sedation (*n *= 96), that is, remifentanil, combined with propofol when required. Inclusion criteria were age ≥ 18 years, start of mechanical ventilation within the previous 24 hours, anticipated requirement of mechanical ventilation for a further 48 to 72 hours, and requirement of both analgesia and sedation. Remifentanil treatment was given for a maximum of 10 days. If patients were not extubated by the end of Day 10, treatment was replaced with a regimen in accordance with current clinical practice at the investigational site. Follow-up of these patients was censored at 10 days (21% conventional arm vs. 8% remifentanil arm). The total ICU follow-up was 28 days. ICU length-of-stay, time of start weaning and of extubation, plus all study medication were recorded.

The UltiSAFE study was performed in accordance with the EU Note for Guidance on Good Clinical Practice and the Declaration of Helsinki. Written informed consent/assent was obtained from all patients or from their legally authorized representatives. Ethics committees and required health authorities of each participating centre approved the study protocol.

### Model structure

A micro-simulation Markov model was used to calculate the costs of remifentanil-based sedation (RS) versus conventional-based sedation (CS) in ICU patients requiring mechanical ventilation from a hospital perspective. The time horizon of the model mimics the clinical study and is 28 days, and the cycle length is one hour, meaning that every hour patients may move to a different health state. The model simulates individual patient histories, containing the time a patient spends in each health state.

**Figure 1:**
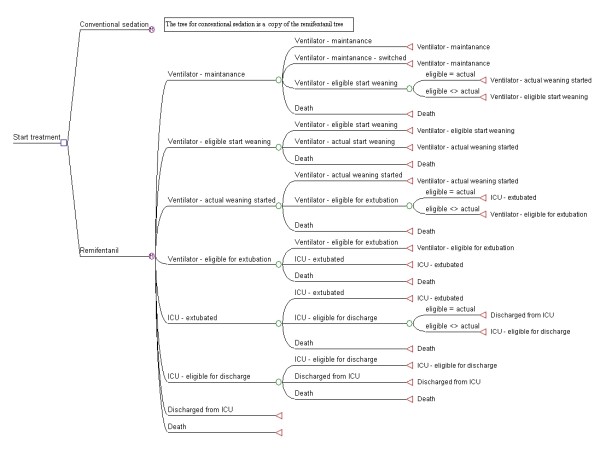
**Model outline**.

In the model, see Figure 1, we distinguish eight health states: 1) Mechanical Ventilation - maintenance; 2) Mechanical Ventilation - eligible to start weaning; 3) Mechanical Ventilation - weaning started; 4) Mechanical Ventilation - eligible to extubate; 5) Post-extubation; 6) Post-extubation - eligible for discharge ICU; 7) Discharged from ICU (final state); 8) Death (final state).

In principle, all patients move through states 1 to 7 in sequence, unless they die (this may occur at any time), thus the transition probabilities used in the model determine when a patient moves to the next state and not if the patient moves. However, for a certain percentage of patients the time between becoming eligible for a transition and the actual transition is zero, meaning that not all patients enter into states 2, 4 and 6. For example, a patient might get extubated immediately after being eligible for extubation and thus will not stay in state 4) "Mechanical Ventilation eligible to extubate" for any length of time.

The model is the same for the RS group and the CS group, with one exception: patients in the RS group may switch to the CS group during the maintenance phase.

### Transition probabilities

The transition probabilities where derived using time-to-event analyses. Often the non-parametric Kaplan-Meier curves are used for such time-to-event analyses; for this study, however, we used Weibull functions to fit the data. This parametric approach allows extrapolation of the data beyond the observation period. This was especially important for the time on mechanical ventilation, since all patients still on MV after 10 days were censored in UltiSAFE.

Within the trial, patients in the maintenance phase could either move to the next phase (eligible to start weaning), die or switch (premature discontinuation), or they could still be on MV at 10 days. So, three time-to-event curves were estimated. For the time-to-'next phase' curve, all patients who had died or switched were considered censored. Likewise, for our Weibull curve of time-to-death all patients who had moved to the next phase or switched were considered censored, and finally, for the time-to-switch patients who had died and moved to the next phase were considered censored.

The Weibull survival curves have the following parameterisation: *S *(*t*) = exp [-((*Lt*)^*p*^)], with L,p > 0. So, for each possible transition from each phase, we estimated the parameters L and p. Additionally, we used a bootstrap procedure to derive the standard errors of L and p, and their correlation [14].

The transition probabilities were derived as follows:

$$tp(t)=1-\frac{S(t)}{S(t-1)}.$$

We did not have actual data from the trial about patients switching from RS to CS. However, we assumed that all patients in the RS group who discontinued the study prematurely while on mechanical ventilation (11%) would switch to the CS group. Thus, the probability of RS patients to switch to CS was estimated by estimating the probability of premature discontinuation in the RS group.

We have assumed in the model that the probability of dying is the same in both groups (that is, we pooled the data from the two treatment groups), since no difference was found in the clinical study. In the maintenance phase, the probability of dying was derived from the Weibull survival curve, in the 'weaning started' and post-extubation phase, constant probabilities were estimated such that the death rate in the clinical study was approximated, since we had too few observed deaths to estimate the survival curves.

Furthermore, we have assumed that the transition probabilities once patients are extubated are the same in both groups for two reasons. First, there is no clinical reason why there would be a difference, once patients are completely weaned from the study drugs, and second, the data showed no difference between groups after extubation.

**Figure 2:**
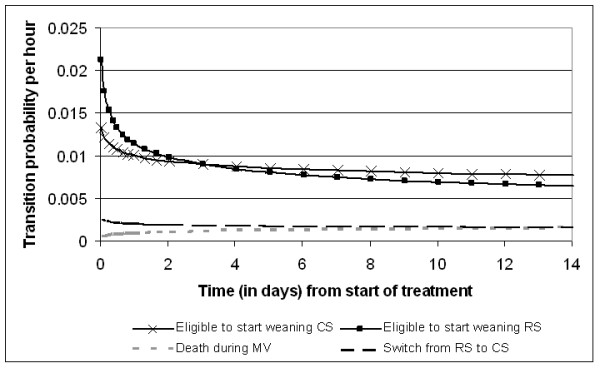
**Hourly transition probabilities from the 'Mechanical ventilation - maintenance' state**.

**Figure 3:**
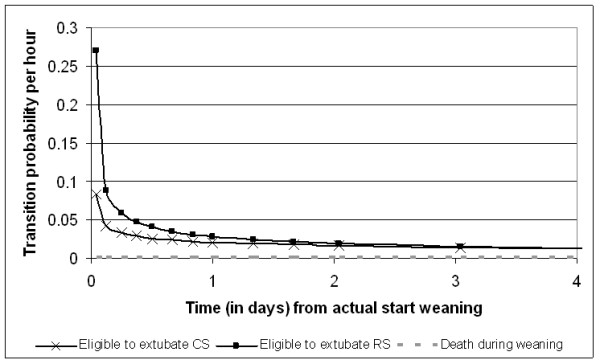
**Hourly transition probabilities from the 'Mechanical Ventilation - weaning started' state**.

**Figure 4:**
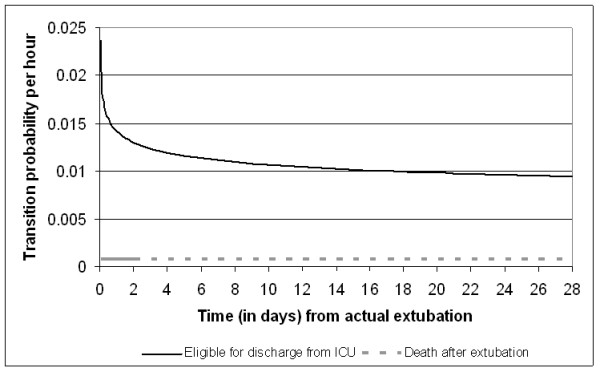
**Hourly transition probabilities from the 'Post-extubation' state**.

**Figure 5:**
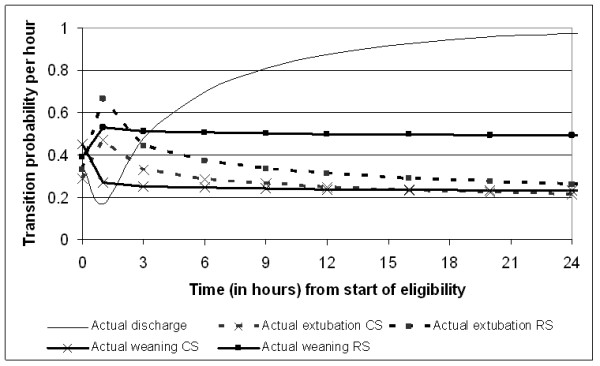
**Hourly transition probabilities from the 'eligible for ...' state (state 2 to 3, state 4 to 5, and state 6 to 7)**.

Figures 2, 3, 4 and 5 present the point estimates for the hourly transition probabilities used in the model. It is clear that most transition probabilities are time-dependent, only the probability of dying during weaning and post-extubation are constant over time.

### Costs

Costs were estimated from the perspective of the hospital with 2006 as the reference year. There are two important cost-components in the model: cost of sedation and cost per day on ICU.

To calculate cost of sedation for both study groups, study drug consumption was derived from UltiSAFE. Every change in dosage was registered, with time, which allowed us to calculate exactly the total dosage per patient per day of every drug involved. We related this to the health state of the patient, that is, before weaning had started (State 1 and 2) and during the weaning phase (State 3 and 4). This way, we derived the average dosage per patient per day per health state per treatment group (see Table 1), with the associated standard error. For the purposes of this model, this was translated into hourly costs, using Dutch costs per milligram as paid by the three hospital pharmacies included in our micro costing study. This resulted in sedation costs per hour before the start of weaning in the CS group of €1.30 (SE 0.13) and in the RS group of €7.47 (SE 0.27). During the weaning phase these sedation costs are €0.41 (SE 0.12) and €3.85 (SE 0.41), respectively.

**Table 1:** Average dosage per patient per day (in mg) of sedatives and analgesics per treatment group and costs per mg (2006 costs)

	Cost per mg (€)	Control group		Remifentanil group	
			
		Before start weaning (mg)	During weaning (mg)	Before start weaning (mg)	During weaning (mg)
		Mean (SE)	Mean (SE)	Mean (SE)	Mean (SE)
Morphine	0.02	71.7 (17.8)	46.5 (24.6)	1.0 (0.4)	2.4 (0.9)
Midazolam	0.035	194.2 (36.2)	12.9 (4.7)	45.9 (22.2)	0.0 (0.0)
Fentanyl	0.35	0.8 (0.1)	0.3 (0.0)	0.0 (0.0)	0.0 (0.0)
Lorazepam	0.04	2.9 (0.7)	0.1 (0.1)	0.0 (0.0)	0.0 (0.0)
Sufenta Forte^®^	25	0.05 (0.0)	0.0 (0.0)	0.0 (0.0)	0.0 (0.0)
Propofol	0.02	1,057.4 (107.0)	381.8 (125.9)	1,627.6 (129.3)	662.2 (154.5)
Remifentanil	7.71	0 (0)	0 (0)	18.8 (0.7)	10.3 (1.2)

Mg, milligram; SE, standard error.

For the cost per day on ICU, an extensive micro-costing study was done separately [13]. Data were collected as to allow the estimation of cost per day either with or without mechanical ventilation. The micro-costing study was conducted in three hospitals (one university hospital, two general hospitals) in The Netherlands for 2006, from a hospital perspective. No ethical approval was required for this micro-costing study. Data on resource use were collected for individual patients on the ICU. Direct costs that were included comprised diagnostics, consumables, hotel and nutrition, and labour. For each of these items, resource use was determined and multiplied by the corresponding unit prices for 2006. The estimates for indirect costs (overhead and capital) were based on the annual accounts 2005 and divided by the direct costs. Thus, indirect costs were allocated to patients using a marginal mark-up percentage. Further details of this micro-costing study are published elsewhere [13]. The average costs per ICU day with mechanical ventilation and without mechanical ventilation were estimated at €2,106 (SE €102) and at €1,645 (SE €107), respectively.

### Model outcomes

The focus of our analysis is the difference in total costs per patient between the two groups. To arrive at the total average costs per patient, we combined the number of hours each patient spent in each health state with the costs per hour of ICU stay (either with or without MV, depending on the health state) and the costs per hour of sedation (either before or after weaning has started). Furthermore, we also report length-of-stay on the mechanical ventilator and in the ICU. These results are reported for the whole patient group, as well as for the subgroup where weaning (that is, transition to State 3) had started within 72 hours.

### Sensitivity analysis

We addressed the uncertainty of our model outcomes through a probabilistic sensitivity analysis. All input parameters were varied by drawing from their probability distribution. For most input parameters, a normal distribution was assumed, though for a few Weibull parameters a lognormal distribution was used. For each set of two Weibull parameters (L and p), we drew first L, and conditional on this value the p. For each new set of input parameters, the model was run to estimate the costs and outcomes. This was then repeated a large number of times (here 1,500), each resulting in new outcomes. From this we derived the confidence interval around our model outcomes.

## Results

The model shows that in the RS group the average total 28-day costs were €15,626 versus €17,100 with CS, meaning a difference in costs of €1,474 (95% CI -2,163, 5,110) (Table 2). When taking all uncertainties about the model input into account, we find that the probability of costs being saved when using remifentanil is 79%. These cost savings are explained by the reduced LOS. On average, patients stay on the ICU for 8.5 days in the CS group, versus 7.6 days in the RS group, leading to a reduction of 0.9 day (95% CI -0.7, 2.6) Based on the probabilistic sensitivity analysis, we estimated that the probability that RS leads to a reduced length-of-stay on the ICU is 89%. When looking at the time on mechanical ventilation (MV), it becomes clear that the reduction in length-of-stay on the ICU is fully due to a reduction in time on MV (see Table 2).

**Table 2:** Mean length-of-stay and costs per treatment group

	RS	CS	Difference (CS-RS)	95% CI	P(diff > 0)
Length of stay ICU	7.6	8.5	0.9	(-0.7;2.6)	89%
Length of stay MV	5.0	6.0	1.0	(-0.8; 2.9)	88%
Costs (€)	15,579	17,064	1,485	(-2,224; 5,194)	79%

RS, remifentanil-bases analgo-sedation; CS, conventional analgo-sedation; CI, confidence interval; diff, difference; ICU, intensive care unit; MV, mechanical ventilation.

When we limit the model results to the subgroup, we see that though the costs and LOSs are lower for each group, the differences between the two treatment groups are approximately the same (Table 3). However, the uncertainties are now smaller, with the probability of costs being saved when using remifentanil being 90%.

**Table 3:** Mean length-of-stay and costs per treatment group for on-label subgroup

	RS	CS	Difference	95% CI	P(diff >0)
			(CS-RS)		
Length of stay ICU	5.1	5.9	0.8	(-0.3; 2.0)	93%
Length of stay MV	2.3	3.2	0.9	(-0.3; 2.2)	94%
Costs (€)	9,807	11,319	1,512	(-1,034; 4,058)	90%

RS, remifentanil-bases analgo-sedation; CS, conventional analgo-sedation; CI, confidence interval; diff, difference; ICU, intensive care unit; MV, mechanical ventilation.

To check the external validity of our model we compared the results of the model to the results of the UltiSAFE trial. In the trial, a median LOS on MV was observed of 5.1 and 3.9 days for CS and RS, respectively. This is slightly higher than the median LOS on MV found with the model: 5.0 and 3.7 days for CS and RS, respectively.

## Discussion

This study showed that using a remifentanil-based sedation reduces costs compared to conventional sedation. The current economic evaluation is based on a model, allowing extrapolation beyond the moment of censoring (at 10 days MV) as it was defined in the clinical study. In that study, after three days still 59% of the patients in the CS group were intubated and 42% in the RS group, even though the intention was to include patients with an expected duration of ventilation of 24 to 72 hours. For this reason, also a subgroup analysis was included for patients that started weaning within the first 72 hours. The reduction of LOS and costs with remifentanil for the subgroup was similar to that for the whole population.

In the clinical study, after 10 days 21% of patients were still intubated in the CS group versus 8% in the RS group. It is clear that the lack of observation of time of extubation leads to uncertainty. We have, therefore, included an extensive probabilistic sensitivity analysis to address this uncertainty. From this we found that there is 79% certainty that using remifentanil-based sedation is indeed cost-saving when considering all patients, and 90% when limiting the analysis to the subgroup.

Commonly in drug trials, a 95% certainty is required before we allow a statement that a new treatment is more effective than the comparator. However, in health economics such strong risk aversion is not common. Some health economists have even argued that it is most rational to base the decision about introduction solely on the expected value [15], but even for risk averse decision makers, 79% probability of being actually cost-saving will be judged as favourable. For comparison, a technology with a 50% probability that its total incremental cost-effectiveness ratio (ICER) is below £20,000 to £30,000/QALY is seen as cost-effective in the UK [16]. Often an ICER of $50,000 US/QALY is also cited by health economics as threshold value - again, at a 50% likelihood [17].

In our model we included the transition to death, based on the deaths that occurred in the clinical study. However, data on deaths were scarce, so, consequently, the transition probabilities to death are surrounded by much uncertainty. But since the death rate was the same in the two treatment groups, any changes in these probabilities will have a very limited effect on the outcome.

To date, no similar economic evaluations have been published. Most studies looking at the costs of remifentanil have focussed on the costs of sedation and analgesics of remifentanil and its alternatives. Only a few have addressed all relevant costs, for example, an open-label randomized study in Germany comparing remifentanil/propofol (*n *= 39) versus midazolam/fentanyl (*n *= 33) [18]. The total costs for these groups were €1,712 versus €1,729. The additional costs of the remifentanil regime were compensated for by lower costs for physicians and nurses.

The perspective used for the cost calculations was that of the hospital. The question may be raised to what extend our results would change had we adopted a societal perspective. In this study, such a perspective would only have changed the drug costs. In the hospital perspective, the costs per mg are based on the prices hospitals pay after discounts. In the societal perspective, the prices before discounts should have been used. Since there was no discount for remifentanil versus large discounts for the other analgesics and sedatives, adopting a societal perspective would have been in favour of the RS arm.

Of course, the model outcomes can only be as good as the input. Since most of our input was derived from the UltiSAFE study, it is important to discuss its findings in relation to our results. The main result of UltiSAFE was that the treatment effect of remifentanil was time-dependent, that is, on Days 1 to 3 patients in the RS group were 1.86 times more likely to be extubated than in the CS group (*P *= 0.018), while no difference was observed during Days 4 to 10 (rate ratio 0.98; *P *= 0.951) [11]. Though our study used Weibull time-to-event curves instead of Cox's proportional-hazards models as in the clinical study, we can still recognize this time-dependency in Figure 2. Here, the transition probability to start weaning is much higher for RS than CS during the first two days, while slightly lower after three days.

Additionally, the strengths and limitations of UltiSAFE should be mentioned as they also apply to the current study. UltiSAFE was not blinded, which may have caused biases. However, the purpose of the study was not to compare two treatments, but two rather different sedation regimens applied in 'real life'. While an inclusion criterion of the study was an anticipated two to three days of mechanical ventilation, after three more days 60% of the patients were intubated. This caused the study to be underpowered, which also impacts the degree of uncertainty around our model estimates of costs and LOS.

It is important to realize that the current cost-consequence study was limited to the parameters evaluated in the main study UltiSAFE. For example, ventilator associated pneumonia and other ICU acquired infections have not been taken into account in the current study.

From the literature it is clear that patients on mechanical ventilation are at an increased risk of developing pneumonia (VAP, ventilator-associated pneumonia). Due to the sample size of UltiSAFE, data on this adverse event were not collected as part of the clinical study on which we based our model input. VAP is the most prevalent infection acquired on ICU; the VAP frequency reported in various studies ranges from 8 to 28% [19].

These studies also show varying results regarding the risk per day on MV. While one study showed a constant risk of 1% per day [20], another study showed a decreasing hazard, going from 3% risk per day at Day 5, to 2% at Day 10 and 1% at Day 15 on MV [21]. The differences in results can mostly be explained by differences in populations being studied [19]. Due to this uncertainty, we opted for not considering these adverse events.

It is possible that inclusion of these might lead to averted cases of VAP in the RS group. Since various studies have reported that VAP increases LOS on MV and on the ICU [22,23], excluding VAP in our model may have led to a conservative estimate of the cost savings due to remifentanil. However, it is possible that some patients in the UltiSAFE as well as in the micro-costing study suffered from VAP, and in that case, some of the increased costs and LOS due to VAP may have been implicitly included in our results.

Conversely, inclusion of ICU acquired infections could also have a negative impact on the cost-consequences of remifentanil. Recently a retrospective case-control study was published that showed that remifentanil discontinuation is an independent predictor of ICU acquired infections [24]. This mechanism has been found in animal studies where morphine withdrawal caused immunosuppression resulting in an increased risk of infection [25,26]. However, it is not clear to what extent this is remifentanil-specific. Given that the animal studies involved morphine it seems likely that ICU patients receiving morphine are also at risk for post-discontinuation infections. Whether this is also true for patients receiving fentanyl is unknown at this time.

Overall we can conclude that more data are needed in order to incorporate ICU acquired infections (including VAP) into a cost-consequence analysis of remifentanil.

Another parameter that was not explicitly studied in the UltiSAFE is the occurrence of acute withdrawal syndrome after opioid discontinuation. In the literature the occurrence of withdrawal syndrome has been reported, though little is known about the frequency of withdrawal syndrome [27,28]. In one retrospective study, patients with an ICU stay of more than seven days were studied for the occurrence of withdrawal syndrome [27]. Of the 28 patients included, 32% developed this syndrome. There was no difference between patients receiving fentanyl or morphine. No studies have been done in patients with shorter ICU stays. In the UltiSAFE study, withdrawal syndrome was reported in only one patient, in the RS group. Clearly larger studies are required to come to a meaningful conclusion on what the probability of withdrawal syndrome is after opioid-discontinuation and whether this probability differs between remifentanil and other opioids.

Additionally, pain resulting from remifentanil discontinuation has been reported [29]. However, this was in the context of a double blind controlled trial of remifentanil versus fentanyl for analgesia based sedation in the ICU. In that study remifentanil patients who experienced pain did so for significantly longer during extubation, post-extubation and post-treatment. This is explained by the rapid offset of the analgesic. However, the authors suggest that in clinical practice, where the clinician is aware of this issue, proactive pain management can avoid this problem. In the UltiSAFE study, three patients in the RS group received morphine or fentanyl during the weaning phase, so it is likely that the costs associated with pain after remifentanil discontinuation are, at least to some extent, already incorporated in our data.

One of the issues with any clinical and cost-effectiveness study is that of generalisability of results to other countries. The UltiSAFE clinical study was performed in Dutch hospitals, and the control group was treated according to Dutch guidelines. As a result, a variety of drugs was used in this group, which may not all be used in other countries. For example, the proportion of patients treated with fentanyl versus morphine can vary. If the reduction in MV found in the UltiSAFE study is explained by a relatively large proportion of patients in the control group using morphine (59%), then the projected cost-savings may not be achieved in a setting where the current analgesic of choice is fentanyl.

However, in other countries studies have been performed with remifentanil in ICU patients where the control group was treated differently than the UltiSAFE.

In a randomized, open-label study remifentanil plus midazolam (*n *= 57) was compared to midazolam plus fentanyl or morphine (*n *= 48) in ICU patients expected to require MV for at least four days [30]. In the control group, 62% of patients received midazolam with fentanyl, 15% received midazolam with morphine and 23% received midazolam alone. In this study the time on MV was, on average, 147 hours in the comparator group versus 94 hours in the remifentanil group, that is, a reduction of 53 hours (36%, *P *= 0.033).

Another randomized, open label study compared remifentanil plus propofol (*n *= 39) to midazolam plus fentanyl (*n *= 33) in post-operative ICU patients expected to require MV for 12 to 72 hours [18]. The time on MV was, on average, 24.2 hours in the control group versus 20.7 hours in the remifentanil group, that is, a reduction of 3.5 hours (14%, *P *< 0.05).

While the patient populations in these studies and the UltiSAFE study are not fully comparable, especially with regards to the expected duration of MV at the time of inclusion, all studies show a clear reduction in MV time in the remifentanil group, both when only fentanyl was used in the control group and when both fentanyl and morphine could be used. Thus, it seems that the impact of different sedation/analgesic regimes on the reduction of MV and thus potential cost-savings is limited.

Finally, we would like to point out that the estimated savings of remifentanil-based sedation represent potential savings: Only if the hospital can use the freed resources (staff and increased ICU-capacity) it can exploit this potential. Furthermore, this analysis was performed in The Netherlands and its results cannot be directly transferred to other countries without necessary adjustments, for example, for country specific relative costs.

## Conclusions

Our modelling study showed that compared to conventional sedation, remifentanil-based sedation decreases the overall costs of an ICU stay and the average ICU length-of-stay.

## Key message

• The higher medication costs of remifentanil-based sedation are compensated by the savings due to decreased ICU LOS, leading to overall cost savings.

## Abbreviations

CS: conventional analgesia and sedation; ICER: incremental cost-effectiveness ratio; ICU: intensive care unit; LOS: length-of-stay; MV: mechanical ventilation; RS: remifentanil-based analgo-sedation; VAP: ventilator-associated pneumonia.

## Competing interests

iMTA (MA, LH and ST) received a research grant from GSK for the economic evaluation. The department of Intensive Care, Erasmus MC (JB) received a research grant from GSK for the clinical study.

## Authors' contributions

MA, LH and JB contributed to the design of the study. MA developed the model and performed the statistical analysis of the clinical trial data. LH was responsible for the design of the micro-costing sub-study. LH and SST were responsible for data collection and data analysis for the sub-study. JB was involved in the data collection for the sub-study. MA drafted the manuscript. All authors were involved in revising the draft manuscript. They all read and approved the final manuscript.
